# Rational design of mutations that change the aggregation rate of a protein while maintaining its native structure and stability

**DOI:** 10.1038/srep25559

**Published:** 2016-05-06

**Authors:** Carlo Camilloni, Benedetta Maria Sala, Pietro Sormanni, Riccardo Porcari, Alessandra Corazza, Matteo De Rosa, Stefano Zanini, Alberto Barbiroli, Gennaro Esposito, Martino Bolognesi, Vittorio Bellotti, Michele Vendruscolo, Stefano Ricagno

**Affiliations:** 1 Department of Chemistry, University of Cambridge, Cambridge CB2 1EW, UK; 2 Department of Chemistry and Institute for Advanced Study, Technische Universität München, Lichtenbergstraße 4, D-85748 Garching, Germany; 3 Dipartimento di Bioscienze, Università degli Studi di Milano, 20133 Milano, Italy; 4 Wolfson Drug Discovery Unit, Centre for Amyloidosis and Acute Phase Proteins, University College London, London NW3 2PF, UK; 5 Dipartimento di Scienze Mediche e Biologiche, Università di Udine, 33100 Udine, Italy; 6 Dipartimento di Scienze per gli Alimenti, la Nutrizione e l’Ambiente, Università degli Studi di Milano, 20133 Milano, Italy; 7 Science and Math Division, New York University Abu Dhabi, Saadiyat Island, Abu Dhabi, UAE; 8 CIMAINA and CNR Istituto di Biofisica, c/o Dipartimento di Bioscienze, Università degli Studi di Milano, 20133 Milano, Italy

## Abstract

A wide range of human diseases is associated with mutations that, destabilizing proteins native state, promote their aggregation. However, the mechanisms leading from folded to aggregated states are still incompletely understood. To investigate these mechanisms, we used a combination of NMR spectroscopy and molecular dynamics simulations to compare the native state dynamics of Beta-2 microglobulin (β2m), whose aggregation is associated with dialysis-related amyloidosis, and its aggregation-resistant mutant W60G. Our results indicate that W60G low aggregation propensity can be explained, beyond its higher stability, by an increased average protection of the aggregation-prone residues at its surface. To validate these findings, we designed β2m variants that alter the aggregation-prone exposed surface of wild-type and W60G β2m modifying their aggregation propensity. These results allowed us to pinpoint the role of dynamics in β2m aggregation and to provide a new strategy to tune protein aggregation by modulating the exposure of aggregation-prone residues.

Protein misfolding and aggregation is a widespread phenomenon associated with the intrinsic properties of polypeptide chains^1^,^2^, indeed, it has been observed that the cross-β structure characteristic of amyloid aggregates often represents the most thermodynamically stable state of polypeptide chains^3^,^4^. Thus, at least at typical cellular concentrations, proteins can be thermodynamically metastable in their native states and do not aggregate only because of the presence of high kinetic barriers^3^,^4^. It is therefore crucial to uncover the strategies that proteins adopt to remain soluble and escape aggregation *in vivo*, and shedding light on the design principles against aggregation would allow a better control of this process^5^,^6^.

Protein aggregation mechanisms can be identified from the analysis of the many amyloidogenic mutants that have been characterized in recent years, including those of transthyretin, lysozyme, β2m, and gelsolin^5^,^6^. Known mutations in these proteins destabilize their native states, often by increasing their dynamics and flexibility^7^,^8^,^9^,^10^,^11^. When destabilized, native states can undergo larger structural fluctuations, or even unfolding, thus favoring the formation of non-native interactions and, ultimately, the deposition into amyloid aggregates^11^.

To determine the molecular origins of these processes, we compare here wild type β 2m and its W60G mutant, to investigate the correlation of thermodynamic stability and conformational flexibility *versus* aggregation propensity. β 2m is a single-domain protein, characterized by a seven-stranded β -sandwich fold typical of the immunoglobulin domain family; β -strands within the protein are named from A to G (Fig. 1A)^12^. β 2m is the light chain of the major histocompatibility complex (MHC) class I^12^,^13^,^14^. While β 2m is highly stabilized by the interactions within the MHC class I^15^, when β 2m is released as a monomer, it may turn into the etiological agent of dialysis-related amyloidosis, a condition triggered by renal impairment and the subsequent chronic accumulation of abnormally high concentrations of β 2m in body fluids. β 2m deposits in joints, bones and muscles, lead to movement impairment, bones fragility and articular pain^16^,^17^.

Recent efforts in elucidating the molecular determinants of β 2m aggregation have highlighted the complex role played by W60, an evolutionary-conserved, solvent-exposed residue. *In silico* simulations of wild-type β 2m suggested that W60 is heavily involved in intermolecular interactions^18^, therefore the non-natural mutation to Gly (W60G) was prepared, yielding to a variant with unexpected properties^19^. In particular, the (unfolding) thermodynamic stability of this mutant is higher than that of the wild-type β 2m, with Δ G°(H_2_O) 22.2 ±  2.0 and 27.6 ±  3.3 kJ mol^−1^, for wild-type and W60G variants, respectively^19^. While both wild-type and W60G aggregate under strongly denaturing conditions (pH 2.5), W60G shows low aggregation propensity using the standard protocol at pH 7.4 and 20% TFE, conditions under which wild-type β 2m aggregates abundantly^19^,^20^. A wealth of data indicates that the DE loop, where W60 is located, is under backbone geometrical strain in wild-type β 2m^18^,^21^,^22^,^23^. The introduction in the DE loop of a residue endowed with higher conformational freedom, such as Gly, relieves the local conformational strain and results in a β 2m variant thermodynamically more stable in solution, with a decreased aggregation propensity^19^,^24^. The above observations prompted us to select wild-type β 2m and its W60G variant as a well-suited system to investigate, besides protein thermodynamic stabilities, other mechanisms that may tune protein aggregation propensity.

To gain further understanding into the molecular bases of the different solution properties displayed by wild-type and W60G forms, we characterized the equilibrium distribution of conformations for the two variants. We employed NMR solution chemical shifts measured under native conditions in combination with replica-averaged metadynamics (RAM) simulations^25^,^26^,^27^. RAM simulations allow integrating the physico-chemical knowledge of a system (*i.e.* the interactions among its atoms as defined in molecular force fields) with the knowledge derived by experimental measurements (*i.e.* chemical shifts), to provide an atomistic description of the Boltzmann distribution of structures that satisfy the experimental data and the maximum entropy principle^28^. Comparison of the resulting ensembles shows how the W60G mutation decreases the overall aggregation propensity by increasing the average β -structure content and by perturbing the overall dynamics of surface residues. To test whether a better average protection of aggregation-prone surface residues can actually be key in regulating protein aggregation, we designed surface mutations aiming to either decrease the aggregation propensity of wild-type β 2m, or increase that of the W60G variant. We found that the designed mutants do not cause any relevant structural rearrangement in the protein core, nor they alter β 2m thermodynamic stability, but display striking different aggregation propensities. Thus, the approach presented here helps to decouple different molecular determinants contributing together to determine a high aggregation propensity, this strategy may be used to understand and modulate the aggregation properties of other folded proteins without affecting their structures and thermal stabilities.

## Results

### NMR chemical shift assignment of the W60G mutant

**Figure 1:**
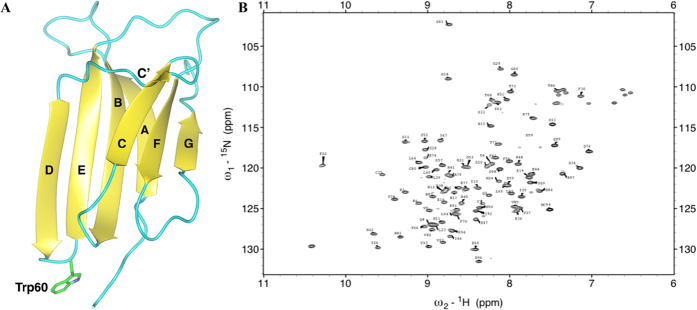
NMR assigment of β2m. (**A**) Ribbon representation of the crystal structure of human β 2m (pdb code 2YXF) with β -strands labeled according to standard nomenclature. W60 is shown as sticks. (**B**) HSQC [^1^H, ^15^N] spectrum recorded at 11.7 T (500.13 MHz for 1H), 310K, of [U-^13^C, U-^15^N W60G β 2m] 0.5 mM dissolved in 70 mM phosphate buffer at pH =   6.6 and 100 mM NaCl.

Wild-type β 2m was already characterized in terms of ^1^H, ^15^N and ^13^C backbone and side chain assignment^29^, whereas for the W60G only ^1^H resonances and amide nitrogens were previously attributed^19^. Here we extended the NMR analysis to the full backbone, as well as to Cβ and Hα , and in favorable cases to Hβ . The ^15^N-^1^H HSQC spectrum at 310 K (Fig. 1B), shows 108 (96 belonging to HNs) of the 110 peaks expected. The missing amide peaks belong to K58 and S88, which were not observed also in the wild-type type form due to unfavorable local exchange broadening^30^. In fact only at very low protein concentration (less than 50 μ M), where the presence of dynamic dimers is scarce, these two peaks are visible. Moreover A15 and Y66 NHs overlap in the HSQC spectrum acquired at 500 MHz. The sequential backbone assignment was obtained using heteronuclear 3D triple-resonance experiments acquired on ^13^C, ^15^N uniformly labeled sample. The assignment percentage for C’, Cα , Cβ were 93%, 97%, 86%, respectively. 95% of Hα and only 23% of Hβ were identified through (H)CCH-TOCSY spectra.

### Native state dynamics of β2m

We determined the conformational properties of wild-type and W60G β 2m using NMR chemical shifts and molecular simulations in the RAM framework^25^,^26^,^27^. In RAM the sampling of the conformational space is enhanced by employing bias-exchange metadynamics^31^, while the quality of the force field is improved by including the information content of equilibrium experiments, *i.e.* NMR chemical shifts^25^,^26^, within the framework of the maximum entropy principle^28^.

**Figure 2:**
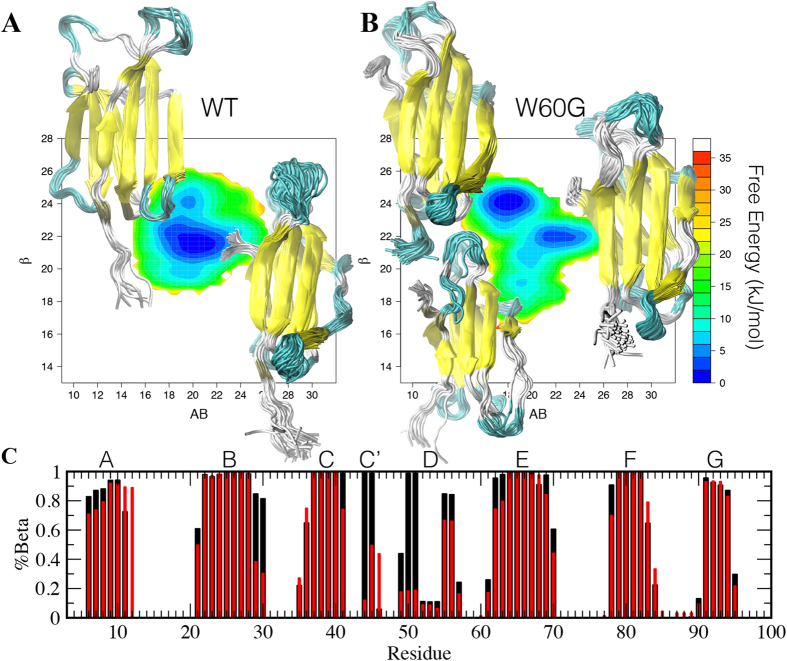
β2m RAM Ensembles. Free energy surfaces (in kJ/mol) for wild-type (**A**) and W60G β 2m (**B**) as a function of the side chains rotameric state, AB, and the antiparallel β -structure content (β ). (**C**) β -structure populations for wild-type (red bars) and W60G (black bars) β 2m. The seven β -strands building the protein fold are identified by A through G labels.

Using the RAM approach we obtained a converged sampling, resulting in a free energy landscape within a statistical uncertainty of less than 2 kJ/mol for free energies up to 20 kJ/mol (Figs S1 and S2). The free energy surfaces for the wild-type and W60G β 2m variants, as a function of the side-chain rotamer distribution (AlphaBeta collective variable, AB, see Methods) and the anti-parallel β -structure content (AntiBetaRMSD collective variable, β , see Methods), are shown in Fig. 2A,B. The free energy surfaces indicate that W60G hosts on average a higher content of β -structure than the wild-type protein. By closer inspection, the main differences in the β -sheet populations (β ) are localized in the B, C’ and D strands (Fig. 2C). These differences shows how the local relaxation of the strain of the backbone in position 60 propagates along the D strand up to C’ due to its reduced freedom on one side and through space to the C-terminal of the B strand on the other. Wild-type β 2m shows a single well-defined minimum at β  =  22 and AB =  20, and a low-populated minimum at higher β -sheet content (β  =  24), compatible with a fully structured D strand. Conversely, W60G shows two minima of comparable energy, one at β  =  24 and AB =  18, and the other at β  =  22 and AB =  22; both minima are close to the two minima of the wild-type protein (Fig. 2). The effect of the W60G mutation on the dynamics of β 2m is also reflected by the reduced structural fluctuations in the 25–31 and 40–60 regions (B,C,C’ and D strands) as shown in Fig. S3.

### Analysis of sequence and structural properties leading to protein aggregation

**Figure 3:**
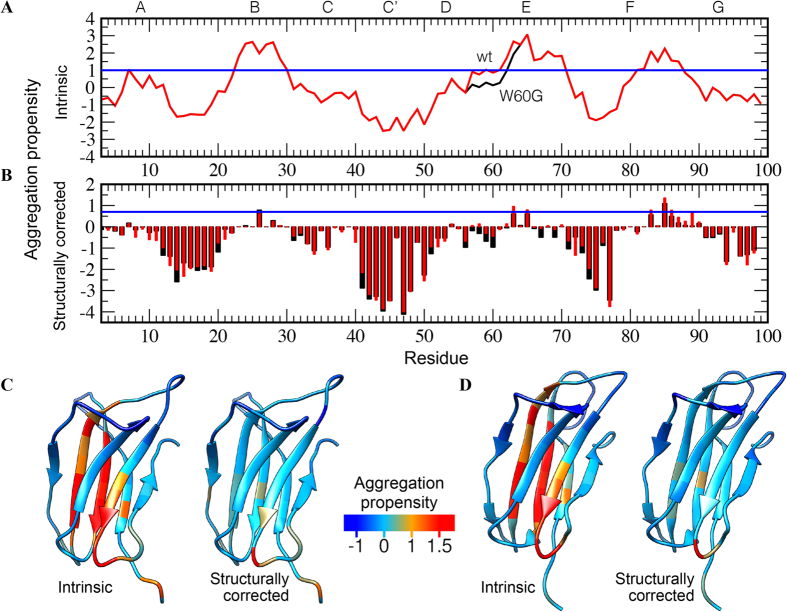
Predicted aggregation properties of β2m wild-type and W60G variants. (**A**) Intrinsic aggregation profiles of the wild-type (red) and W60G (black) variants. (**B**) Structurally corrected profiles calculated as ensemble averages for β 2m wild-type (red bars) and W60G (black bars). In (**A**,**B**) values larger than 1 denote aggregation-prone regions. (**C**) Projection on the structure of wild-type β 2m of the intrinsic profile (left) and the structurally corrected profile (right). The color code is blue for aggregation-resistant residues and red for aggregation-prone residues. (**D**) Same as (**C**) for the structure of W60G β 2m.

Wild-type and W60G are characterized by significantly different aggregation properties. While the wild-type protein can form fibrils under both neutral (pH 7.4) and acidic (pH 2.5) conditions, the mutant yields fibrils only under denaturing (pH 2.5) conditions^19^,^20^. Such difference was explained in terms of the different thermodynamic stability of the two variants. NMR relaxation studies under conditions far from those favoring aggregation confirm that the wild-type species exhibits a much more pronounced association propensity than the W60G mutant^30^. However, additional insight can be obtained by analyzing the amyloidogenic properties of β 2m in terms of sequence- and structure-based properties. The intrinsic and structurally corrected aggregation propensities were assessed using the CamSol method (Fig. 3A,B)^32^, which is highly accurate in predicting mutation-induced changes in protein solubility, a property often strongly correlated with aggregation^33^. In the present work we used aggregation propensity scores that are the inverse of the CamSol solubility scores (so that higher scores represent higher aggregation propensity) and, in order to analyze the RAM ensemble, we have defined a ‘total structurally-corrected score’ (see Methods). The latter can be used to predict the tendency to aggregate of individual conformations of the ensemble; it is calculated from the structurally corrected CamSol profile in the same way the CamSol intrinsic score is calculated from the intrinsic profile^32^. The ensemble average of the total structurally corrected scores for the two β 2m variants is − 0.59 ±  0.01 and − 0.66 ±  0.01, for the wild-type and W60G, respectively. Such scores are in keeping with the experimental aggregation data, *i.e.* wild-type β 2m is more aggregation prone than W60G β 2m under native conditions.

The intrinsic and the structurally corrected profiles are represented in Fig. 3C,D, color-coded on the structures of wild-type and W60G. Aggregation-prone residues are mostly located in the B, E strands, and at the C-terminal of the F strand (Fig. 3A). These β -strands are the central strands in the two β 2m β -sheets; the B and E strands are flanked by the A and D strands (in the 4-stranded β -sheet), while the F strand is surrounded by the C and G strands (in the 3-stranded β -sheet) (Fig. 1A). It is noteworthy that all these flanking β -strands consistently prove less aggregation-prone than the central ones, according to both the intrinsic and the structurally corrected CamSol profiles (Fig. 3B), an observation that may be interpreted as a strategy to protect the protein from unwanted aggregation. Furthermore, at least half of the aggregation-prone residues point towards the protein core, thus being excluded from the chance of unwanted interactions with other proteins under native conditions (Fig. 3B). The analysis recognizes the A strand and the CD and EF loops as the protein protective regions. Interestingly, the strong aggregation propensity of the naturally occurring truncated β 2m variant, devoid of the first six residues (Δ N6)^34^, can be rationalized in the above context; in fact, by shortening the protective A strand, the C-terminal residues of the B strand, which are the most aggregation-prone, become solvent exposed. By the same token, one may also explain the behavior of D76N-β 2m, the naturally occurring mutant responsible for a severe systemic amyloidosis^10^. In fact, the ‘fatal’ mutation leading to the most amyloidogenic isoform of β 2m so far described occurs in the protective EF loop.

**Figure 4:**
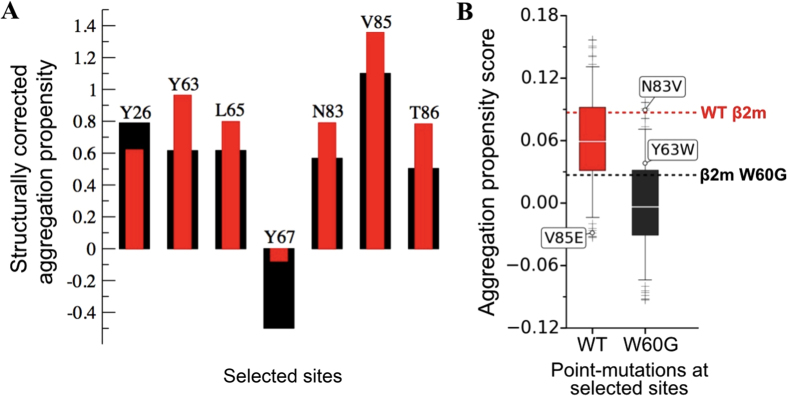
Rational design of β2m mutants. (**A**) Aggregation-prone residues (intrinsic score >  1) for which the protective effect of the W60G mutation is the highest, defined as those residues with the largest change in the structurally-corrected score between the wild-type (red) and W60G (black) β 2m. (**B**) Predicted aggregation propensity scores calculated using CamSol for all possible amino acid substitutions at the sites shown in panel A in the wild type (left box-plot in red) and in the W60G variant (right box-plot in black). The red and black dashed lines correspond to the aggregation propensity score of the WT and the W60G variant, respectively. Aggregation-protective and aggregation-promoting mutations selected for experimental validation are flagged. V85E and N83V mutations have been chosen as those with the largest predicted effect, while Y63W was chosen as a weakly aggregation promoting mutation. In each box the horizontal line denotes the median of the distribution; whiskers extend from the 5th to the 95th percentile of the distribution and boxes from the lower to the upper quartile.

The structurally corrected aggregation propensity profile for W60G shows an increase in the average protection of the aggregation-prone residues (Figs 3B,D and 4). Such an effect can be linked to the reduced strain at site 60 that propagates thorough the D strand in the 4-stranded β -sheet to the opposite β -sheet (Fig. 3D); indeed W60G shows on average a higher β -structure content distributed along all its β -strands (Fig. 2A–C) together with reduced fluctuations (Fig. S3).

### Rational design of mutants with different aggregation propensities

The above analysis suggests then that the role of the W60G mutation in reducing aggregation is not exclusively due to stabilization of the protein. In particular, by optimizing the geometry of the D strand through the W60G mutation, all the aggregation-prone residues of the central β -strands, in both β -sheets, become on average more protected (Fig. 3). These observations suggest that by specifically modulating the average surface properties arising from the native state dynamics, it may be possible to tune the aggregation properties of β 2m.

To test the role of dynamics on β 2m native state aggregation we searched for mutations able to decrease the aggregation propensity of wild-type β 2m, on one hand, or increasing the aggregation propensity of W60G, on the other, without affecting their thermodynamic stabilities and structures. In Fig. 4A the residues with an intrinsic score higher than 1, for which the W60G mutation results in a large variation (> 0.2) of the structurally corrected score with respect to the wild-type are highlighted. These seven residues are all located in the central strands of both β -sheets. Table S1 reports the amino acid substitutions, known within the β 2m protein family, that occur at such selected residue sites^35^; the sequence data highlight that none of these sites show strong residue conservation. Mutations of residues 65 and 67, in the E strand, have already been studied^36^, even though under different experimental conditions, showing that residues in this β -strand are critical for aggregation. Mutations in the F strand have not yet been investigated. By calculating the change in the protein intrinsic score upon all the possible single mutations at these seven sites (Fig. 4B), we suggest that the V85E β 2m variant should reduce the wild-type aggregation propensity, while two alternative specific mutations in the W60G β 2m variant (W60G-Y63W and W60G-N83V) are expected to weakly (the former) or largely (the latter) increase the W60G aggregation propensity. Indeed, the score of V85E β 2m is lower than that of W60G, the one of W60G-N83V is comparable to that of the wild-type while W60G-Y63W is slightly larger than that of W60G (Fig. 4B). All three mutations are located on the protein surface, and two of them are also naturally occurring in vertebrates other than human; hence they should cause little perturbation to the protein structure and stability. It is noteworthy that the effects of surface mutations introducing or removing surface charges, do not correlate simply with aggregation propensity: the V84D and Y86E mutations in acylphosphatase from *S. solfataricus* do not protect the protein from unfolding and aggregation^37^. Furthermore, a D to N systematic scanning in β 2m indicated that the removal of a negative charge triggers measurable effects in aggregation propensity only when the D to N mutation occurs at a specific site^38^.

### Structural characterization of three rationally-designed β2m surface mutants

The three β 2m surface mutants that we rationally designed (V85E, W60G-N83V, W60G-Y63W) were produced as recombinant proteins, purified to homogeneity, crystallized, and their structures determined at high resolution (1.75 Å, 1.49 Å, 1.70 Å, for V85E, W60G-Y63W, and W60G-N83V, respectively). Although the three crystals belong to the same space group, unit cell edges differ and crystal packing is distinct for each of the three mutants (see methods). The three structures display electron density of excellent quality along the polypeptide chains. Data collection and refinement statistics are reported in Table S2.

Inspection of the refined X-ray crystal structures shows that all three surface mutations are well tolerated in the β 2m fold, and backbone conformational changes are relegated to loops (Table S3). Indeed, the largest structural deviations in the three mutants occur far from the mutation sites, at the AB loop (residues 12–21) that is typically in an open conformation in the crystal structures of isolated β 2m, while it is closed over the rest of the β -sandwich when β 2m is part of the MHC class I and in few structures of monomeric β 2m^39^,^40^,^41^. Notably, the AB loop in a closed conformation is consistently observed in solution NMR structures^42^. In particular, for the crystal structures here reported of the W60G-Y63W and V85E mutants a closed AB loop conformation was observed; by contrast, the wild-type β 2m, W60G and W60G-N83V 3D structures exhibit an open AB loop conformation. All these data suggest that the AB loop can easily adjust to different conformations, which, in the case of the present mutants, is likely the result of convenient molecular packing in the crystals.

**Figure 5:**
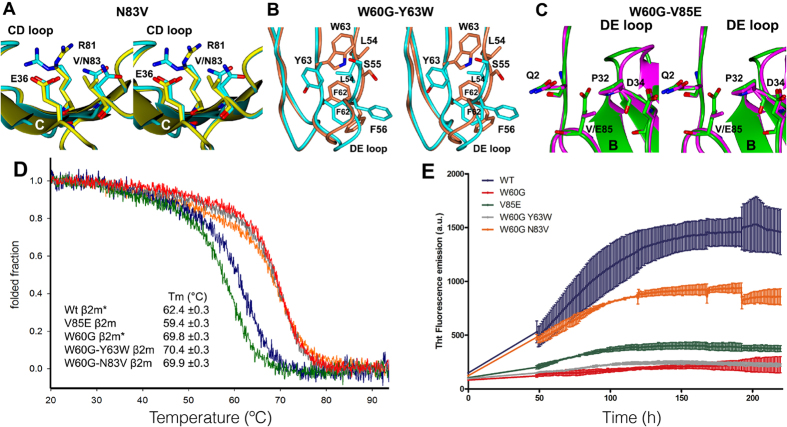
Structure, stability and aggregation of the β2m variants. (**A**) The superimposed structures of the W60G and W60G-V83N mutants are shown in cyan and yellow, respectively. (**B**) Zoom of the (**D** and **E**) strands: the superposed structures of the W60G and W60G-Y63W are shown in cyan and coral, respectively. (**C**) Superimposed structures of wild-type β 2m and the V85E variant, at the mutation site, shown in magenta and green, respectively. (**D**) Thermal unfolding of wild-type β 2m, of the W60G variant and of the three surface mutants, monitored by far-UV CD at 202 nm. In the table the measured Tm are reported (*the Tm of wild-type and W60G variants have been previously reported^23^). (**E**) Comparison of the kinetics of fibril formation of the various mutational variants of β 2m analyzed in this work monitored by fluorescence in a thioflavin T assay.

Detailed inspection shows that the structure of the W60G-N83V mutant is perfectly superimposable onto that of W60G β 2m. In fact, in W60G the side chain of N83, which is located on the F-strand, although surrounded by two charged residues (E36 and R81), does not establish any hydrogen bond with the neighboring residues; thus, the N83V substitution leaves both the conformation of residue 83 and of the surrounding side chains unaffected (Fig. 5A).

Residue 63 lies in the E strand, in close vicinity of the DE loop (residues 57–60). The Y63W mutation triggers just a minor reorientation of the DE loop backbone, but is responsible of extensive, though local, side chain reorganization. In the structure of the W60G-Y63W mutant the bulkier W63 side chain flips away from the DE loop and towards the D-strand, compared to what is observed in W60G. Such movement has two consequences: (1) the shift of S55, F56, L54 and F62 side chains away from W63, and (2) the DE loop moves about 2 Å towards residue 63 (Fig. 5B).

The comparison of the wild-type β 2m and the V85E mutant structures reveals that the mutation does not elicit local rearrangements in the FG loop where E85 is located, nor in the surrounding residues. The 3D-structure of V85E mutant displays modified conformations in the DE and C’D loops; however, given that both regions are far from the mutation site, these differences may be due to the specific crystal packing (Fig. 5C).

### Stability of the rationally-designed β2m surface mutants

The stability of all three mutants described above was evaluated by monitoring their thermal denaturation through far-UV CD measurements. In keeping with the goals set (*i.e.* designing mutants that maintained the same thermodynamic stability either of wild-type β 2m or of the W60G mutant) and with the outcomes of the crystallographic analyses, none of the three mutations was found to alter dramatically the protein stability. Upon V85E mutation, the wild-type β 2m stability was slightly impaired, with a T_m_ value decreased by about 3 °C relative to wild-type β 2m. The negative charge of E85 lies 4.5 Å away from the carboxylate of D34, the electrostatic repulsion between these two residues may contribute to the slightly lower stability observed for the V85E mutant compared to the wild-type protein (Fig. 5C). Conversely, introduction of the Y63W and N83V mutations (added to the W60G mutation) did not vary significantly the T_m_ value recorded for the W60G mutant (Fig. 5D).

### Aggregation measurements of the rationally-designed β2m surface mutants

To further test the effects of the three designed mutations, the aggregation propensity of the V85E, W60G-Y63W, and W60G-N83V mutants was tested at pH 7.4 and in 20% TFE, conditions under which wild-type β 2m aggregates readily while the W60G mutant yields only small amounts of aggregates^19^,^43^. Figure 5E shows that the V85E mutation, despite being slightly less stable than the wild-type protein, almost totally abolished wild-type β 2m aggregation-propensity, and that the W60G-N83V variant presented a markedly increased aggregation trend with respect to W60G β 2m. The W60G-Y63W variant displayed the same very low aggregation propensity as the W60G mutant, suggesting that mutations should be chosen in order to maximize the difference of the total average CamSol score (Fig. 4B).

## Discussion

As protein aggregates are often thermodynamically stable in living organisms^1^, the avoidance of the aggregation process has been optimized through evolution by means of multiple strategies^44^. While efficient protection mechanisms work at the cellular level by regulating protein homeostasis^45^,^46^, many strategies to prevent aggregation concern the properties of the native states of proteins, including a high thermodynamic and kinetic stability, limited conformational fluctuations, and the presence of structural motifs that are incompatible with the cross-β assembly^1^,^4^,^47^. As folded proteins are metastable with respect to aggregation^3^, mutation that destabilize the folded states can dramatically increase the rates of aggregation. Furthermore, mutations that change the accessibility to a high free energy state, without changing the overall stability of the protein native state, are enough to enhance aggregation^11^,^48^. Conversely, mutations that introduce structural protections on edge strands play a protective role independently of the overall protein stability^37^.

Here we investigated the different roles played by stability and dynamics in protein aggregation using human β 2m, a protein that, when released as a monomer and accumulated in patients’ sera, may become the etiological agent of dialysis-related amyloidosis, forming amyloid deposits in joints, bones and muscles^17^. Esposito *et al.* reported previously an engineered β 2m variant bearing the W60G mutation, which was found to be thermodynamically very stable (Cm increased by 0.7 M GdHCl) and resistant to aggregation relative to the wild type β 2m^19^,^20^. To elucidate the molecular determinants underlying such distinct properties, we characterized the native state fluctuations of the wild-type protein and of the stable W60G mutant using NMR chemical shifts and RAM simulations, and analyzed the aggregation properties of the resulting conformational ensembles.

Our simulation results show that the W60G mutation greatly reduces the flexibility of the protein native state (Fig. S3), and increases the overall content of residues adopting β -structure (Fig. 2), in keeping with previous experimental data^19^,^20^,^30^. Moreover, our analysis shows that β 2m hosts three aggregation-prone regions located essentially in three β -strands that are central in the β 2m two β -sheets, *i.e*. strands B, E and F (Fig. 1A), and that in these regions those residues that are exposed on the surface are better protected on average in the W60G variant than in the wild-type (Fig. 3). The differences in the native state dynamics together with the similarity of their sequence-based intrinsic scores, explains the different behaviors observed under native and denaturing conditions, *i.e.* while both protein variants aggregate under denaturing conditions (pH 2.5), W60G shows little aggregation propensity under the standard aggregation protocol, at pH 7.4 and 20% TFE^19^,^20^.

To probe the role played by the residues in the three central β -strands in determining β 2m aggregation propensity, and to further investigate the protective effect of the W60G mutation, we designed three mutations aimed to modulate β 2m aggregation trends under native conditions, while leaving β 2m stability and structure unaltered. To this aim, we focused on mutations of protein surface residues that turned out to be better protected in the W60G ensemble compared to the wild-type protein. The V85E single site β 2m mutant, together with the W60G-Y63W and the W60G-N83V double mutants were accordingly designed and produced in recombinant form. Consistent with such rational approach, we found that all three surface mutants maintain unaltered the β 2m core structure (Fig. 5 and Table S2) and display fold stabilities very similar to wild-type β 2m (for V85E), or to W60G (for the two double mutants), respectively. Conversely, analysis of their aggregation properties indicates that surface mutations can indeed modulate β 2m aggregation propensity. The V85E mutation, on the F strand, abrogates completely wild-type β 2m amyloidogenicity, under the tested conditions, despite being slightly less stable than the wild-type; the W60G-N83V mutant (residue 83 falls on the F strand) gains a non-negligible aggregation propensity relative to W60G without any difference in protein stability. We cannot rule out that the fibrillar aggregate may form following a different aggregation pathway compared to wild-type β 2m as it was shown in details for mutants of acylphosphatase from *S. Solfataricus*^49^. However in such case this phenomenon was coupled to very different thermodynamic stability of the native protein^49^. The W60G-Y63W double mutant, on the other hand, displays an aggregation trend comparable to W60G and in agreement with the small change in the aggregation score (Fig. 4B), indicating that this conservative mutation cannot encode noticeable changes of the aggregation propensity.

β 2m hosts extensive aggregation regions on protein surface (Fig. 3), this being rooted in its sequence and structure that are specifically optimized to be part of the MHC class I. In particular, the aggregation-prone residues W60 and Y63 are buried at the interface between β 2m and the MHC class I heavy chain^19^, consistent with the observation that protein interface residues are typically aggregation prone^50^. On the contrary, the F strand, which has not been previously studied, plays a pivotal role in the aggregation propensity of β 2m, and it is solvent-exposed in MHC class I. In particular, site 85 in human β 2m bears a dangerously aggregation-prone Val residue. Such apparent design error, however, could be reconciled with a recent observation^15^ whereby the MHC class I heavy chain stabilizes markedly the β 2m fold.

In summary our results provide an explanation for the resistance to aggregation associated with the W60G mutation of β 2m. This mutation not only brings a significant increase in the thermodynamic stability of the protein, but also generates a more regular and less flexible overall structure in which the surface aggregation-prone regions are better protected. We suggest that such second feature is primarily responsible for the reduced aggregation propensity of this variant. Based on these observations, we showed that the aggregation propensity of β 2m can be tuned by mutating surface residues without affecting its structure and stability, thus highlighting the crucial role played by the dynamics of surface residues in protein aggregation. Our results elucidate a mechanism whereby the aggregation propensity of a protein can be modulated by mutations that change the average protection of its aggregation-prone surface residues with minor effects on the structure and stability of the protein itself.

## Materials and Methods

### Multidimensional NMR spectroscopy

All data were recorded on a U-^13^C, U-^15^N labeled W60G β 2m sample 0.5 mM in phosphate buffer 70 mM, NaCl 100 mM, pH 6.6, at 310K with a Bruker Avance 500 and with a Varian INOVA spectrometers operating at proton frequency of 500 and 800 MHz, respectively. Proton chemical shifts were referenced to 2,2-Dimethyl-2-silapentane-5-sulfonate sodium salt, DSS, whose resonance was set to 0.0 ppm. ^13^C and ^15^N chemical shifts were referenced indirectly to DSS, using absolute frequency ratio^51^. Sequence specific assignment were achieved using [1H, 15N]-HSQC and [1H, 13C]-HSQC together with 3D triple-resonance experiments, in particular by using the [15N, 1H]- HNCA/[15N, 1H]- HN(CO)CA for the identification of patterns of sequentially linked spin systems and 3D [15N, 1H]- HNCACB/[15N, 1H]-HN(CO)CACB pair of experiments, which allowed the identification of the amino acid types. 3D (H)CCH-TOCSY was used for the assignment of Hα and Hβ resonances.

The spectra were processed with Topspin 2.0 (Bruker Biospin) and analysed into the Sparky (T. D. Goddard and D. G. Kneller, SPARKY3, University of California, San Francisco) framework.

### Molecular dynamics simulations

All the simulations in the present work were performed using GROMACS compiled with PLUMED and ALMOST. The system was simulated using the Amber03W force field in explicit TIP4P05 water. A time step of 2 fs was used together with LINCS constraints. van der Waals and short-range electrostatic interactions were cut-off at 0.9 nm while long range electrostatic was treated with the Particle Mesh Ewald method and a mesh size of 0.12 nm. The canonical ensemble was enforced by keeping the volume fixed and by thermosetting the system with the Nosé-Hoover thermostat at 310K. The starting conformation for the wild-type was taken from the 1BMG X-Ray structure while for W60G was taken from the NMR structure 2VB5. The structures were protonated and solvated with 11500 water molecules in a dodecahedron box of 360 nm^3^ of volume.

### Replica-averaged Metadynamics

RAM simulations were performed using chemical shifts as replica-averaged restraints and bias-exchange metadynamics. The bias-exchange metadynamics approach combines replica exchange^52^ with metadynamics, in which several metadynamics simulations are performed in parallel on different replicas of the system, each replica biasing a different collective variable (CV). Exchanges between the replicas are attempted periodically according to a replica-exchange scheme. Four replicas of the system were simulated in parallel at 310 K with a restraint applied on the average value of the CamShift back-calculated NMR chemical shifts where the force constant is set to 24 kJ/(mol ppm^2^).

Each of the four replica is bias along one of the following four collective variables, CVs: the anti-parallel β content (the ‘β ’ CV), the parallel β -sheet content (the ‘Pβ ’ CV), the AlphaBeta collective variable defined over all the chi1 angles for the hydrophobic side-chains (the ‘AB’ CV) and the AlphaBeta collective variable defined over all the phi and psi backbone dihedral angles of the protein (the ‘bbAB’ CV). The choice of the secondary structures CVs was guided by the fact that the protein is a full beta protein. The to AlphaBeta CVs are designed to sped up the fluctuations of the backbone and the side-chains irrelevant of their specific conformation. Gaussians deposition was performed with an initial rate of 0.125 kJ/mol/ps, a bias-factor of 8 and with σ values set to 0.1, 0.04, 0.16 and 0.25, for β , Pβ , AB and bbAB, respectively. Furthermore, in order to limit the extent of accessible space along each collective variable and correctly treat the problem of the borders, we set the bias as constant outside a defined interval for each CV; intervals were set to 19–29, 3–7, 14–30 and 47–71 for the four CVs, respectively. Each replica have been evolved for 200 ns, with exchange trials every 50 ps.

The sampling of the four replicas was used to generate a four-dimensional free energy landscape, as a function of the before mentioned CVs, where a set of microstates is identified by dividing the four-dimensional CV-space into a homogeneous grid of small dimensional hypercubes and their free energy is obtained using a standard weighted histogram analysis. Bi-dimensional FESes are obtained integrating out the variables not showed.

### Cloning, mutagenesis, expression and purification

Recombinant vectors pEX containing genes encoding for W60G-Y63W, W60G-N83V variants, flanking NdeI/XhoI sites were purchased from Eurofins MWG Operon^©^. An empty vector pET-29b and the vectors carrying the β 2m variants were digested with NdeI and XhoI enzymes (New England Biolabs (UK) Ltd^©^) to obtain a linearized pET29 vector and the isolated genes. Wild-type β 2m, W60G-Y63W and W60G-N83V genes were inserted in pET-29b vector with a ligation reaction (Thermo Scientific Rapid DNA Ligation Kit, Life Technologies^©^). pET-29b-WTβ 2m plasmid was used as template DNA for Site-Directed Mutagenesis to obtain the V85E mutant. The following primers were used: 5′ -GCC TGC CGT GTG AAC CAT **GAG** ACT TTG TCA CAG CCC-3′ , 3′ -GG GCT GTG ACA AAG **TCT** CAT GGT TCA CAC GGC AGG C-5′ . All mutants were expressed and purified as previously reported^34^.

### Crystallization and X-ray structure determination

All mutants were crystallized with sitting drops technique. Lyophilized W60G-Y63W, W60G-N83V and V85E β 2m mutants were solubilized in ddH_2_O at a concentration of 10 mg/mL, 8.5 mg/mL and 8 mg/mL respectively at 20 °C. β 2mW60G-Y63W crystals were obtained in 0.1 M HEPES pH 7.5, 20% (w/v) PEG 10K buffer (Crystal Screen HT^TM^, Hampton). The crystals were cryoprotected in 0.08 M HEPES pH 7.5, 18% PEG 10K, and 20% (v/v) glycerol and flash-frozen in liquid nitrogen. W60G-N83V crystallized in 0.1 M MES pH 6, 21% PEG 4K, 15% glycerol, 0.2 M ammonium acetate, while V85E mutant was crystallized in 0.1 M ammonium acetate pH 5.5, 30% PEG 4K, 15% glycerol and 0.2 M ammonium acetate. The crystals were flash-frozen using mother liquor as cryoprotectant.

X-ray data were collected at ESRF (European Synchrotron Radiation Facility of Grenoble–France) at the ID23-2 beam line. The diffraction data were analyzed and processed using MOSFLM^53^, the crystal symmetry was then verified by POINTLESS^54^ and the intensities were merged with SCALA^55^. The crystal structure was determined by molecular replacement using MOLREP^56^ using the structure of W60V β 2m mutant (pdb code: 2Z9T) as searching model^22^. The model molecules placed in the asymmetric unit were subjected firstly to a rigid-body refinement and then to a restrained refinement using Phenix Refine in the Phenix program suite^57^. Manual model building, addition of water molecules and ligands were then performed using the molecular graphic software Coot^58^.

All three mutants crystallized in C2 space group however the underlying crystal packing is different: the two double mutants (W60G-Y63W, W60G-N83V) have one molecule in the asymmetric unit (AU); however, the different conformation of the AB loop determines distinct intermolecular interactions in the crystals. Unusually, the AU of the V85E structure contains two molecules. These different crystal packings determine some changes in the protein regions involved in the intermolecular interactions, however, these differences are not deemed relevant for the discussion on the properties of the three mutants.

### Circular dichroism spectroscopy

Thermal stability experiments, performed in the far-UV region, were carried out on a J-810 spectropolarimeter (JASCO Corp., Tokyo, Japan) equipped with a Peltier system for temperature control. The protein concentration was 0.1 mg/mL in 50 mM sodium phosphate pH 7.4. The temperature ramp measurements were recorded from 20 to 95 °C (temperature slope 50 °C/hour) in a 0.1 cm path length cuvette and monitored at 202 nm wavelength. T_m_ was calculated as the first-derivative minimum of the traces. Spectra before and after unfolding ramp were recorded (260–190 nm).

### Aggregation assays

Samples (100 μ L) of recombinant variants W60G, V85E, W60G-Y63W, W60G-N83V, and wild-type β 2m at 100 μ M in 50 mM phosphate buffer (pH 7.4), 100 mM NaCl, 20% trifluoroethanol and containing 10 μ M Thioflavin T (ThT) (SIGMA), were incubated at 37 °C in Costar 96-well black- wall plates sealed with clear sealing film (4TITUDE) and were subjected to 900 rpm double-orbital shaking. In each well fragmented fibrils of wild type β 2m were added as seeds. Bottom fluorescence was recorded at 15-min intervals (BMG LABTECH FLUOstar Omega). Fluorescence was monitored in three or more replicate tests.

### NMR assignment deposition

1H, 15N and 13C assignments of W60G β 2m have been deposited to Biological Magnetic Resonance Bank with accession code 25809.

### Structure deposition

Atomic coordinates and structure factors for the β 2m mutants W60G-Y63W, W60G-N83V, V85E have been deposited with the Protein Data Bank, with accession codes 5CFH, 5CKA and 5CKG, respectively.

## Additional Information

**How to cite this article**: Camilloni, C. *et al.* Rational design of mutations that change the aggregation rate of a protein while maintaining its native structure and stability. *Sci. Rep.* **6**, 25559; doi: 10.1038/srep25559 (2016).

## Supplementary Material

Supplementary Information
