# Supplementary material for: Rational design of mutations that change the aggregation rate of a protein while maintaining its native structure and stability
Source: Sci Rep. 2016 May 6;6:25559. doi: 10.1038/srep25559 (PMC4858664; doi:10.1038/srep25559)
Supplement: Supplementary Information [file srep25559-s1.doc]

**Rational design of mutations that change**

**the aggregation rate of a protein while maintaining**

**its native structure and stability**

Carlo Camilloni^1,2,*^, Benedetta Maria Sala^3^, Pietro Sormanni^1^, Riccardo Porcari^4^, Alessandra Corazza^5^, Matteo De Rosa^3^, Stefano Zanini^5^, Alberto Barbiroli^6^, Gennaro Esposito^5,7^, Martino Bolognesi^3,8^, Vittorio Bellotti^4^, Michele Vendruscolo^1^ and Stefano Ricagno^3,*^

*^1^Department of Chemistry, University of Cambridge, Cambridge CB2 1EW, UK.*

*^2^Department of Chemistry and Institute for Advanced Study, Technische Universität München, Lichtenbergstraße 4, D-85748 Garching, Germany*

*^3^Dipartimento di Bioscienze, Università degli Studi di Milano, 20133 Milano, Italy.*

*^4^Wolfson Drug Discovery Unit, Centre for Amyloidosis and Acute Phase Proteins, University College London, London NW3 2PF, UK*

*^5^Dipartimento di Scienze Mediche e Biologiche, Università di Udine, 33100 Udine, Italy.*

*^6^Dipartimento di Scienze per gli Alimenti, la Nutrizione e l’Ambiente, Università degli Studi di Milano, 20133 Milano, Italy.*

*^7^Science and Math Division, New York University Abu Dhabi, Saadiyat Island, Abu Dhabi, UAE*

*^8^ CIMAINA and CNR Istituto di Biofisica, c/o Dipartimento di Bioscienze, Università degli Studi di Milano, 20133 Milano, Italy.*

^*^Corresponding authors: Carlo Camilloni, carlo.camilloni@ch.tum.de; Stefano Ricagno, stefano.ricagno@unimi.it

**Supporting Figures and Tables**

**Figure S1:** Convergence of the free-energy profiles along the four selected collective variables for the WT ensemble.

**Figure S2:** Convergence of the free-energy profiles along the four selected collective variables for the W60G ensemble.

**Figure S3:** Average backbone fluctuations for WT (red) and W60G (black) ensembles.

**Table S1: Sequence variability through vertebrates in seven sites relevant for aggregation.**

| **Residue (WT)** | **Known Substitutions** |
| --- | --- |
| Y26 | F/H/L/R |
| Y63 | H/K/N/Q |
| L65 | S/T |
| Y67 | F/H/Q/S/T |
| N83 | K/Q/R/S/T/**V** |
| V85 | D/**E**/G/I/L/M/N/S/T |
| T86 | A/G/K/N/Q/S |

List of the known sequence variability through vertebrates as in (Raimondi et al., 2011) for surface residues with largest change in aggregation propensity upon W60G mutation. Amino acids in bold are those selected in the design process (i.e. WT-V85E and W60G-N83V). The third designed mutation, W60G-Y63W, was not selected among the known substitutions.

**Table S2: Data collection and refinement statistics.**

| **Structure** | **β2mW60G-Y63W** | **β2mW60G-N83V** | **β2mV85E** |
| --- | --- | --- | --- |
| Beam Line | ID29 (ESRF) | ID29 (ESRF) | ID29 (ESRF) |
| Space group | C 1 2 1 | C 1 2 1 | C 1 2 1 |
| Unit cell constants (Å) | a = 93.35, b = 29.09, c = 44.41, β = 113.01° | a = 76.98, b = 28.91, c = 57.32, β = 128.57° | a = 88.58, b = 28.86, c = 87.72, β = 110.11° |
| Resolution (Å) | 25.11 – 1.49  (1.57 – 1.49) | 25.75 – 1.70 (1.79 – 1.70) | 27.71 – 1.75 (1.84 – 1.75) |
| R_merge_ (%) | 7.5 (22.0) | 5.6 (20.1) | 6.4 (32.3) |
| I/σI | 9.6 (4.5) | 12.4 (5.0) | 9.3 (2.9) |
| Completeness (%) | 95.8 (96.5) | 96.7 (97.1) | 97.0 (96.9) |
| Multiplicity | 3.3 (3.4) | 3.5 (3.7) | 3.7 (3.7) |
| Unique reflections | 17435 (2537) | 10677 (1540) | 20777 (3026) |
| Refinement |  |  |  |
| R_work_ (%) | 16.9 | 17.6 | 18.3 |
| R_free_ (%) | 23.0 | 22.5 | 23.2 |
| Number of atoms | 961 | 992 | 1880 |
| Protein | 820 | 894 | 1746 |
| Water | 141 | 71 | 120 |
| Heteroatoms |  | 27 | 14 |
| Ramachandran plot, *n* (%) |  |  |  |
| Most favoured region | 100 | 97.2 | 96.2 |
| Allowed region | 0 | 2.8 | 3.3 |
| Ouliers | 0 | 0 | 0.5 |

aRmerge = Σhkl ΣjIhkl,j - <Ihkl>/ΣhklΣjIhkl,j where I is the observed intensity and <I> is the average intensity.

bRwork = ΣhklFo - Fc/ΣhklFo for all data except 5–10%, which were used for the Rfree calculation.

Values given in parenthesis refer to the high-resolution shell.

**Table S3: Structural similarities between β2m variants.**

|  | **WT _(mon)_** | **WT _(MHC)_** | **W60G** |
| --- | --- | --- | --- |
| **W60G-Y63W** | 0.96Å/ 93 Cα | 0.93Å/ 97 Cα | 0.84Å/ 93 Cα |
| **W60G-N83V** | 0.61Å/ 99 Cα | 1.13Å/ 92 Cα | 0.28Å/ 100 Cα |
| **V85Eb** | 0.81Å/ 92 Cα | 1.03Å/ 98 Cα | 0.91Å/ 94 Cα |

RMSD values calculated from the structural superposition of the three surface mutants (W60G-Y63W, W60G-N83V and V85E) with monomeric wt **β**2m (PDB code 2YXF), displaying AB loop in open conformation; wt **β**2m from an MHC class I complex (PDB code 2BSS), displaying AB loop in closed conformation; and with the structure of the W60G mutant (PDB code 2Z9T).
